# Influence of metabolic syndrome on disease characteristics and activity in inflammatory bowel disease: A retrospective cohort study

**DOI:** 10.1097/MD.0000000000048426

**Published:** 2026-04-24

**Authors:** Pingping Liu, Xue Jing, Lingling Sun, Shijin Wang, Jingli Zhang, Xueli Ding, Lijuan Sun, Beibei Ma

**Affiliations:** a Department of Gastroenterology, The Affiliated Hospital of Qingdao University, Qingdao, Shandong, China; b Department of Pathology, The Affiliated Hospital of Qingdao University, Qingdao, Shandong, China; c Department of Radiology, The Affiliated Hospital of Qingdao University, Qingdao, Shandong, China.

**Keywords:** disease activity, inflammatory bowel disease, metabolic syndrome

## Abstract

The incidence of inflammatory bowel disease (IBD) and metabolic syndrome (MetS) has risen in recent years, but the relationship between the 2 remains understudied. This retrospective cohort study aimed to explore the impact of MetS on disease activity in IBD. Retrospective analysis of 676 consecutive IBD patients treated from January 2023 to April 2024. Study participants were stratified into a control cohort of 531 patients with uncomplicated IBD, and an exposure cohort of 145 patients with IBD and concurrent MetS. Data were collected and analyzed for all 676 patients at baseline and after 1-year follow-up. From an initial pool of 1730 potentially eligible patients, 156 (9%) were identified with MetS (IBD-MetS cohort: 86% ulcerative colitis and 14% Crohn’s disease). Baseline clinical disease activity was significantly higher than in the IBD-only controls, with concomitant elevations in biomarkers including monocytosis, eosinophilia, elevated C-reactive protein and elevated erythrocyte sedimentation rate. At 12-months’ follow-up, the IBD-MetS group showed significantly lower rates of clinical remission (54.48% vs 82.49%) and mucosal healing (24.83% vs 41.05%) compared to the controls. Inflammatory biomarkers remained elevated, consistent with baseline findings. After propensity score-matched analysis, a comparative analysis of 145 matched controls and 145 exposed groups showed that, compared to IBD-only patients, IBD-MetS patients still had significantly lower rates of clinical remission (54.48% vs 77.93%, odds ratio: 0.339, 95% confidence interval: 0.203–0.565, *P* < .001) and mucosal healing (24.83% vs 41.38%, odds ratio: 0.468, 95% confidence interval: 0.283–0.772, *P* = .003). Our findings indicate that MetS is correlated with increased disease activity in IBD. These findings emphasize the need for clinicians to identify and intervene in metabolic syndrome components in IBD patients, ultimately improving quality of life and providing new insights for future IBD-MetS research.

## 1. Introduction

Inflammatory bowel disease (IBD), comprising Crohn’s disease (CD) and ulcerative colitis (UC), is characterized by chronic relapsing inflammation of the gastrointestinal tract.^[1]^ Although its precise etiology remains unclear, evidence points to complex interactions involving genetic susceptibility, environmental or microbial factors, and dysregulated immune responses.^[2]^ CD typically presents with segmental, transmural inflammation that may affect any part of the gastrointestinal tract,^[3]^ whereas UC involves continuous, superficial inflammation confined to the colon.^[4]^ The global incidence of IBD has risen sharply in recent decades, with eastern nations exhibiting the most pronounced increase, driven by economic growth and dietary shifts.^[5,6]^

Metabolic syndrome (MetS) includes abdominal obesity, diabetes, hypertension, hypertriglyceridemia and low high-density lipoprotein (HDL) cholesterol.^[7]^ The development of MetS is associated with insulin resistance and a chronic low-grade inflammatory state. The worldwide prevalence of MetS has shown a marked increase, a trend well exemplified by its sharp rise in China from 13.7% to 31.1% between 2001 and 2017, largely attributable to high-calorie, low-fiber diets and poor lifestyle habits.^[8,9]^

The comorbidity rate between IBD and MetS ranges from 10.6% to 32.7%.^[10]^ These conditions share several pathophysiological features, including immune dysregulation, chronic low-grade inflammation, insulin resistance and gut microbiota alterations.^[11]^ At the molecular level, genes co-expressed in both diseases have been identified.^[12]^ Previous studies have focused on the impact of diabetes or obesity on the disease activity of IBD.^[13,14]^ At the histopathological level, MetS may alleviate IBD.^[15]^ However, patients in the terminal phase of MetS − a state characterized by impaired glucose tolerance and normo- or hypoinsulinemia − present with more severe UC in both clinical manifestations and histopathological features.^[16]^ Furthermore, CD patients with comorbid MetS experience higher hospitalization rates than those with CD alone.^[17]^ Overall, research on how MetS as an integrated entity influences IBD disease activity remains limited. Given the scarcity of data in Asian populations, this study was conducted to investigate the impact of MetS on disease activity in patients with IBD.

## 2. Materials and methods

This retrospective cohort study investigated the impact of MetS on disease activity in IBD. The study protocol was approved by the Medical Ethics Committee of the Affiliated Hospital of Qingdao University (QYFYWZLL30454).

### 2.1. Patient inclusion

Patients with established diagnosis of IBD (encompassing UC and CD) confirmed by standard clinical, endoscopic, and histopathological criteria were included.^[18,19]^ All included patients aged >18 years, had a minimum follow-up duration of >1 year and required either comprehensive electronic medical records or availability for telephone follow-up. The exclusion criteria were as follows: pregnancy; incomplete endoscopic or electronic medical record data; follow-up duration <1 year; history of malignancy within the past 5 years (including gastrointestinal malignancies, lung cancer, breast cancer, thyroid cancer, leukemia, and lymphoma); severe comorbidities (including heart failure, acute coronary syndrome, respiratory failure, acute-phase cerebrovascular disease, hepatic or renal insufficiency, and severe malnutrition); and concurrent autoimmune diseases (including systemic sclerosis, anti-neutrophil cytoplasmic antibody-associated vasculitis).

### 2.2. Assessment

According to the National Cholesterol Education Program Adult Treatment Panel III criteria,^[20]^ a diagnosis of MetS can be made when an individual fulfills at least 3 of the following 5 diagnostic criteria: increased waist circumference (≥102 cm in men and ≥88 cm in women; elevated triglycerides (≥150 mg/ dL or ≥1.69 mmol/L or treatment for high triglycerides; reduced HDL-cholesterol (HDL cholesterol <1.03 mmol/L or <40 mg/dL) in men and <1.29 mmol/L or <50 mg/dL in women, or treatment for low HDL–cholesterol levels; high blood pressure (systolic pressure ≥130 mm Hg and/or diastolic pressure ≥85 mm Hg) or receiving antihypertensive therapy; elevated fasting glucose levels (≥5.6 mmol/L or ≥100 mg/dL) or receiving antidiabetic treatment. In accordance with American Association of Clinical Endocrinology guidelines and China’s practical circumstances, body mass index (BMI) ≥ 25 kg/m^2^ was substituted for the waist circumference criterion.^[21]^

### 2.3. Outcomes

#### 2.3.1. Data collection

Patient data were acquired through either electronic medical record review or telephone follow-up. The general characteristics included age, gender, smoking, and alcohol consumption. The variables related to MetS included BMI, serum triglycerides, serum HDL-cholesterol, fasting blood glucose and blood pressure. The variables related to IBD included disease duration, type of IBD, use of parenteral nutrition, presence of extraintestinal manifestations, baseline medication use and history of IBD-related surgery. The variables related to IBD activity included clinical disease severity, endoscopic disease severity and biomarkers (albumin level, monocyte count, eosinophil count, C-reactive protein (CRP) level and erythrocyte sedimentation rate [ESR]). Based on the Montreal classification, UC patients were categorized by disease extent as E1 (proctitis), E2 (left-sided colitis) or E3 (extensive colitis).^[22]^ The data of extraintestinal manifestations involved the musculoskeletal system, skin, and eyes.^[23]^ The complications of IBD included intestinal fistula, bowel perforation, intestinal obstruction, toxic megacolon, intra-abdominal abscess and perianal abscess.^[4,24]^

#### 2.3.2. Definitions related to disease activity of IBD

Disease activity in IBD was evaluated through standardized clinical scoring systems and endoscopic scoring indices. The modified Mayo score for assessing UC activity comprises 4 components: stool frequency, rectal bleeding, endoscopic findings and physician’s global assessment.^[25,26]^ This score ranges from 0 to 12 points, with ≤2 points (no subscore >1) indicating normal or clinical remission, 3 to 5 points mild activity, 6 to 10 points moderate activity and 11/12 points severe disease activity.^[19]^ The Crohn’s disease activity index is used to assess disease activity in CD,^[27]^ and categorizes disease activity as follows: <150 indicates normal or clinical remission, 150–220 mild activity, 221–450 moderate activity and >450 severe activity.^[18]^ The Mayo endoscopic subscore for UC ranges from 0 to 3, with 0 indicating normal/inactive disease, 1 mild activity, 2 moderate activity and 3 severe activity.^[28]^ The Simplified Endoscopic Activity Score for Crohn’s Disease assesses disease severity, with total scores categorized as follows: 0 to 2 indicates remission, 3 to 6 mild activity, 7 to 15 moderate activity and ≥16 severe activity.^[29]^

### 2.4. Statistical analysis

Descriptive statistics were computed for all variables. The normality of continuous variables was assessed using the Shapiro–Wilk test. Normally or approximately normally distributed variables were summarized as mean ± standard deviation, while skewed variables were reported as median and interquartile range. Categorical variables were expressed as proportions. Missing data (<20%) were multiply imputed (*m* = 5) using R 4.5.0. Pooled results from the imputed datasets are reported. For comparative analyses, group differences were evaluated as follows: the unpaired Student *t* test was used for normally distributed continuous variables, the Mann–Whitney *U* test for skewed continuous variables, and Pearson’s chi-square test for categorical variables. Regression analyses were then performed. Binary logistic regression was employed to examine the dose–response relationship between the number of metabolic syndrome components and clinical remission in IBD. Multiplicative and additive interactions among metabolic syndrome components were also assessed via binary logistic regression. To account for multiple comparisons, the false discovery rate was controlled using the Benjamini–Hochberg procedure. Propensity score matching (PSM; 1:1 nearest-neighbor, caliper = .02) balanced covariates. Statistical significance was set at *P* < .05. Analyses were performed in R v4.5.0.

## 3. Results

### 3.1. Patient population

**Figure 1.**
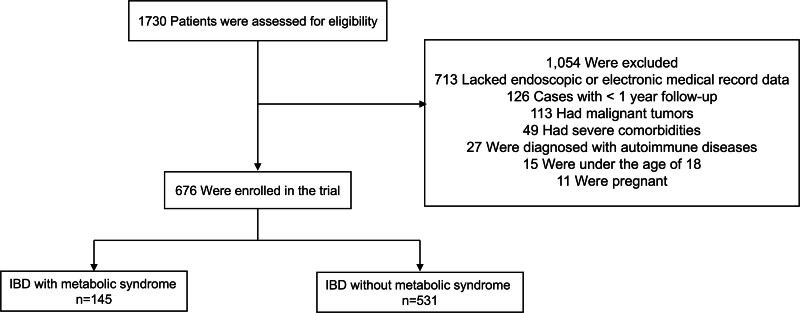
Flowchart of participant selection. IBD = inflammatory bowel disease.

Among the 1730 IBD patients who visited our institution between January 2023 and April 2024, 676 were enrolled based on the selection criteria, while 1054 were excluded: 15 for age <18 years; 113 for a history of malignancy within the past 5 years; 49 for severe comorbidities; 126 for insufficient follow-up duration (<1 year); 27 for comorbid immune-mediated diseases; 11 for pregnancy; and 713 patients for not undergoing colonoscopy, incomplete colonoscopy examinations, missing medical records and unresolved critical data gaps despite telephone follow-up. Among the 1730 IBD patients evaluated, 156 (9%) were identified with MetS (IBD MetS cohort: 86% UC and 14% CD). After applying exclusion criteria, the final analysis included 145 patients in the exposed group (with MetS) and 531 in the control group (without MetS; Fig. 1).

### 3.2. Baseline disease clinical characteristics

We conducted statistical analyses comparing 145 patients in the exposed group with 531 patients in the control group. In terms of the general characteristics, compared to the IBD control group, IBD MetS patients were older (*P* < .001), more likely to be smokers and drinkers (*P* < .001), with no significant difference in gender between the groups (*P* = .149; Table 1). The groups showed no significant differences in IBD duration (*P* = .321), subtype of IBD (*P* = .159), parenteral nutrition (*P* = 1.000), extraintestinal manifestations (*P* = .609), drug exposure (*P* = .533) or surgical history (*P* = .064; Table 1).

**Table 1:** Baseline characteristics of inflammatory bowel disease patients.

Characteristic	Total (n = 676)	IBD MetS (n = 145)	IBD (n = 531)	*P*-value
General characteristics				
Age, mean ± SD	49.56 ± 15.01	58.14 ± 12.20	47.21 ± 14.86	<.001
Gender, n (%)				.149
Female	292 (43.20)	55 (37.93)	237 (44.63)	
Male	384 (56.80)	90 (62.07)	294 (55.37)	
Smoking, n (%)	77 (11.39)	36 (24.83)	41 (7.72)	<.001
Alcohol consumption, n(%)	60 (8.88)	29 (20.00)	31 (5.84)	<.001
Disease characteristics				
Disease duration (yr), mean ± SD	3.75 ± 5.06	4.12 ± 5.27	3.65 ± 5.00	.321
IBD subtype, n (%)				.159
E1	191 (28.25)	51 (35.17)	140 (26.37)	
E2	106 (15.68)	22 (15.17)	84 (15.82)	
E3	318 (47.04)	58 (40.00)	260 (48.96)	
CD	61 (9.02)	14 (9.66)	47 (8.85)	
Extraintestinal manifestations, n (%)	17 (2.51)	5 (3.45)	12 (2.26)	.609
Treatment characteristics				
Parenteral nutrition, n (%)	9 (1.33)	2 (1.38)	7 (1.32)	1.000
Baseline medication, n (%)				.533
No medication history	227 (33.58)	46 (31.72)	181 (34.09)	
5-aminosalicylic acids	369 (54.59)	78 (53.79)	291 (54.80)	
Hormonal agents	28 (4.14)	9 (6.21)	19 (3.58)	
Immunomodulators or biologics	52 (7.69)	12 (8.28)	40 (7.53)	
Surgical history, n (%)	7 (1.04)	4 (2.76)	3 (0.56)	.064
IBD clinical activity, n (%)				<.001
Normal or remission	127 (18.79)	26 (17.93)	101 (19.02)	
Mild	320 (47.34)	50 (34.48)	270 (50.85)	
Moderate	149 (22.04)	42 (28.97)	107 (20.15)	
Severe	80 (11.83)	27 (18.62)	53 (9.98)	
IBD endoscopic activity, n (%)				.320
Normal or remission	31 (4.59)	9 (6.21)	22 (4.14)	
Mild	107 (15.83)	28 (19.31)	79 (14.88)	
Moderate	373 (55.18)	72 (49.66)	301 (56.69)	
Severe	165 (24.41)	36 (24.83)	129 (24.29)	
Biomarkers of activity				
Hypoalbuminemia, n (%)	124 (18.34)	31 (21.38)	93 (17.51)	.286
Monocytosis, n (%)	114 (16.86)	33 (22.76)	81 (15.25)	.032
Eosinophilia, n (%)	45 (6.66)	17 (11.72)	28 (5.27)	.006
Elevated CRP, n (%)	138 (20.41)	42 (28.97)	96 (18.08)	.004
Elevated ESR, n (%)	156 (23.08)	44 (30.34)	112 (21.09)	.019

Categorical variables are presented as n (%); continuous variables are presented as mean ± standard deviation.

CD = Crohn’s disease, CRP = C-reactive protein, E1 = proctitis, E2 = left-sided colitis, E3 = extensive colitis, ESR = erythrocyte sedimentation rate, IBD = inflammatory bowel disease, MetS = metabolic, SD = standard deviation.

### 3.3. MetS components

In comparison to the control group, IBD MetS patients had a higher BMI (*P* < .001), elevated serum triglycerides (*P* < .001), lower serum HDL (*P* < .001), higher fasting blood glucose (*P* < .001) and a greater likelihood of hypertension (*P* < .001; Table 2).

**Table 2:** Characteristics of metabolic syndrome components in inflammatory bowel disease patients.

Characteristic	Total (n = 676)	IBD MetS (n = 145)	IBD (n = 531)	*P*-value
BMI, mean ± SD	23.16 ± 3.33	26.14 ± 3.29	22.35 ± 2.84	<.001
Triglycerides, *M (Q*_1_, *Q*_3_)	1.20 (0.76,1.60)	1.82 (1.41, 2.19)	1.05 (0.68,1.44)	<.001
HDL-C, *M (Q*_1_, *Q*_3_)	1.31 (0.99,1.63)	1.14 (0.89, 1.43)	1.35 (1.03,1.66)	<.001
Fasting glucose, *M (Q*_1_, *Q*_3_)	4.81 (4.33,5.49)	5.63 (4.89, 6.52)	4.66 (4.30,5.27)	<.001
Hypertension, n (%)	97 (14.35)	73 (50.34)	24 (4.52)	<.001

Categorical variables are presented as n (%); continuous variables are presented as mean ± standard deviation or median (first quartile, third quartile).

BMI = body mass index, HDL-C = high-density lipoprotein cholesterol, IBD = inflammatory bowel disease, MetS = metabolic syndrome, SD = standard deviation.

### 3.4. Baseline disease activity

At baseline, there was a significant difference in clinical disease activity between the 2 groups (*P* < .001). Compared to the IBD control group, IBD MetS patients had a higher likelihood of moderate to severe IBD activity. In terms of inflammatory biomarkers, as opposed to the control group, IBD MetS patients demonstrated significantly higher rates of monocytosis (*P* = .032), eosinophilia (*P* = .006), elevated CRP (*P* = .004) and accelerated ESR (*P* = .019). No significant differences were observed between the groups in endoscopic disease activity (*P* = .320) and hypoalbuminemia (*P* = .286; Table 1).

### 3.5. Disease activity and inflammatory marker outcomes at 12-months’ follow-up

After 12-months’ follow-up, regarding disease activity, IBD MetS patients demonstrated a lower clinical remission rate (54.48% vs 82.49%; *P* < .001) and mucosal healing rate (24.83% vs 41.05%; *P* < .001) compared to the control group. They were also more likely to exhibit monocytosis (25.52% vs 12.62%; *P* < .001), eosinophilia (11.03% vs 5.08%; *P* = .009), elevated CRP (23.45% vs 14.88%; *P* = .014), accelerated ESR (27.59% vs 16.01%; *P* = .001) and hypoalbuminemia (15.17% vs 8.66%; *P* = .021; Table 3).

**Table 3:** Disease activity and inflammatory marker outcomes at 12-months’ follow-up.

Variables	Total (n = 676)	IBD MetS (n = 145)	IBD (n = 531)	*P*-value
Clinical remission, n (%)	517 (76.48)	79 (54.48)	438 (82.49)	<.001
Mucosal healing, n (%)	254 (37.57)	36 (24.83)	218 (41.05)	<.001
Monocytosis, n (%)	104 (15.38)	37 (25.52)	67 (12.62)	<.001
Eosinophilia, n (%)	43 (6.36)	16 (11.03)	27 (5.08)	.009
Elevated CRP, n (%)	113 (16.72)	34 (23.45)	79 (14.88)	.014
Elevated ESR, n (%)	125 (18.49)	40 (27.59)	85 (16.01)	.001
Hypoalbuminemia, n (%)	68 (10.06)	22 (15.17)	46 (8.66)	.021

Categorical variables are presented as n (%).

CRP = C-reactive protein, ESR = erythrocyte sedimentation rate, IBD = inflammatory bowel disease, MetS = metabolic syndrome.

### 3.6. Healthcare resource utilization and clinical event outcomes at 12-months’ follow-up

IBD MetS patients had significantly more annual outpatient visits (*P* = .035), higher annual healthcare expenditure [11,514 Chinese Yuan (CNY) vs 9176CNY; *P* = .029], increased relapse-associated readmission rate (23.45% vs 10.17%; *P* < .001) and a greater incidence of IBD-related surgery (4.14% vs 1.13%; *P* = .038) compared to the controls. Despite numerically higher rates of treatment escalation, extraintestinal manifestations and complications in IBD MetS patients compared to the controls, these differences did not reach significance (see Table S1, Supplemental Content 1, Supplemental Digital Content, https://links.lww.com/MD/R741, which illustrates the healthcare resource utilization and clinical event outcomes at 12-months’ follow-up).

### 3.7. Logistic regression analysis of clinical remission rates

Logistic regression analysis revealed that MetS patients had a significantly lower clinical remission rate compared to non-MetS patients (odds ratio [OR] = 0.254, 95% confidence interval [CI]: 0.171–0.378; *P* = .001). After adjusting for age, smoking, alcohol consumption, baseline clinical activity of IBD, and IBD duration and subtype, MetS remained associated with reduced clinical remission (adjusted OR = 0.284, 95%CI: 0.180–0.450; *P* = .001). The 676 IBD patients were reclassified based on the components of MetS. When the exposure factors were hypertriglyceridemia, low HDL-cholesterol, diabetes, hypertension and obesity, the clinical remission rates in the exposed group were 0.329, 0.665, 0.353, 0.411, and 0.588 times those in the control group, respectively. After multivariable adjustment for covariates, patients with hypertriglyceridemia, low HDL-cholesterol, diabetes, hypertension or obesity had significantly lower clinical remission rates, with adjusted odds ratios of 0.342, 0.919, 0.374, 0.478, and 0.671, respectively, compared to their corresponding control groups. Our analysis demonstrated significant associations between hypertriglyceridemia, diabetes, hypertension and IBD disease activity. Collinearity among the independent variables was assessed using variance inflation factors. All variance inflation factors values were below 5 (range: 1.051–1.582), indicating the absence of significant multicollinearity. The False Discovery Rate correction, using the Benjamini–Hochberg procedure, was applied to account for multiple hypothesis testing in the associations evaluated for metabolic syndrome and its components (a total of 6 tests). Statistical significance was determined based on the resulting *q*-values < .05. The key findings were robust to this adjustment, with no change in the overall interpretation (Table 4).

**Table 4:** Crude and adjusted odds ratios of various factors associated with clinical remission.

	N (clinical remission rate [%])		OR (95% CI) *P* (FDR-adjusted q^*^)		
	MetS definition 1	MetS definition 2	Crude	Model 1^†^	Model 2^‡^
MetS					
Yes	145 (54.48)	111 (54.95)	0.254 (0.171, 0.378) .001 (.002)	0.284 (0.180, 0.450) .001 (.002)	0.316 (0.194,0.513) .001 (.002)
No	531 (82.49)	565 (80.71)	1 (reference)		
High serum triglycerides					
Yes	143 (58.74)	142 (59.15)	0.329 (0.221, 0.489) .001 (.002)	0.342 (0.220,0.534) .001 (.002)	0.352 (0.225,0.550) .001 (.002)
No	533 (81.24)	534 (81.09)	1 (reference)		
Low serum HDL-C					
Yes	182 (70.88)		0.665 (0.452, 0.977) .038 (.038)	0.919 (0.602, 1.403) .695 (.695)	
No	494 (78.54)		1 (reference)		
DM					
Yes	179 (61.45)		0.353 (0.242, 0.514) .001 (.002)	0.374 (0.246, 0.569) .001 (.002)	
No	497 (81.89)		1 (reference)		
HTN					
Yes	129 (62.02)		0.411 (0.272,0.621) .001 (.002)	0.478 (0.299, 0.764) .002 (.003)	
No	547 (79.89)		1 (reference)		
Obesity					
Yes	207 (69.57)	78 (64.10)	0.588 (0.406, 0.853) .005 (.006)	0.671 (0.447,1.006) .054 (.065)	0.473 (0.275,0.815) .007 (.008)
No	469 (79.53)	598 (78.09)	1 (reference)		

Data are presented as N (%), where N represents the total number of patients meeting the criteria for each metabolic component, and clinical remission rate (%) indicates the proportion achieving remission. MetS Definition 1 and 2 are based on NCEP ATP III and IDF 2006 criteria, respectively. Model 2 applies the IDF criteria with a BMI cutoff of 28 kg/m^2^ for obesity. Crude and adjusted odds ratios (ORs) with 95% confidence intervals (CIs) are shown. The crude model uses MetS definition 1 without adjustment.

CI = confidence interval, DM = diabetes mellitus, HDL-C = high-density lipoprotein cholesterol, HTN = hypertension, MetS = metabolic syndrome, OR = odds ratio.

* FDR-adjusted *q*-values are provided in parentheses, with *q* < 0.05 denoting statistical significance.

† Model 1 adjusts for age, smoking, alcohol consumption, baseline clinical activity of IBD, IBD duration, and IBD subtype.

‡ Model 2 uses the revised MetS/obesity definition with the same adjustments. All reported *P* values underwent false discovery rate (FDR) correction for 6 tests (Benjamini–Hochberg procedure).

To test the robustness of our findings, we performed a sensitivity analysis by altering the definition of metabolic syndrome from the NCEP Adult Treatment Panel III criteria to the IDF 2006 criteria,^[30]^ simultaneously replacing central obesity with a BMI of ≥ 28 kg/m^2^. With these changes, the number of patients identified with hypertriglyceridemia was 142 (previously 143), with obesity was 78 (previously 207), and meeting the full criteria for metabolic syndrome was 111 (previously 145). Using these revised groupings, we repeated the binary logistic regression analyses with clinical remission as the dependent variable. Consequently, in the obesity analysis, the association with clinical remission, which was non-significant in Model 1 (OR = 0.671, *q* = .065), became statistically significant in model 2 (OR = 0.473, *q* = .008). All other results remained consistent with those from model 1 (Table 4).

Through binary logistic regression, we statistically analyzed both multiplicative and additive interactions among the components of metabolic syndrome. The results indicated no significant multiplicative interactions between any pairwise combinations of metabolic syndrome components (*P* > .05; see Tables S2–S11, Supplemental Content 2–11, Supplemental Digital Content, https://links.lww.com/MD/R741, which illustrates the multiplicative interactions between pairwise combinations of metabolic syndrome components).

In the analysis of additive interactions between pairwise components of metabolic syndrome, statistically significant interactions (*P* < .05) were observed for the pairs of obesity and diabetes, obesity and hypertension, obesity and hypertriglyceridemia, diabetes and hypoalphalipoproteinemia, hypertension and hypertriglyceridemia, hypertension and hypoalphalipoproteinemia, and hypertriglyceridemia and hypoalphalipoproteinemia. However, the point estimates for the measures of interaction were small, and their confidence intervals included the null value (i.e., the 95% CI for relative excess risk due to interaction included 0, for attributable proportion due to interaction (AP) included 0, and for *S* included 1). This indicates that while the observed additive interactions were statistically detectable, their effect magnitudes were weak and potentially lacking in clinical or public health importance. (see Table S12–S14, S18–S21, Supplemental Content 12–14,18–21, Supplemental Digital Content, https://links.lww.com/MD/R741, which illustrates the additive interaction between any pairwise combinations of metabolic syndrome components).

For the additive interaction analysis on the outcome of IBD clinical remission (a favorable outcome), a synergistic interactionon the risk scale for the detrimental outcome (i.e., non-remission) is indicated by relative excess risk due to interaction >0, AP > 0, and *S* < 1. This is because when the outcome is reversed to “non-remission,” the synergy index becomes the reciprocal (*S*’ = 1/*S*), thereby transforming an *S* < 1 in the remission model to an *S*’ > 1 in the non-remission model, which is the conventional indicator for synergy. This interpretation is consistent with methodological guidelines for interaction analysis with protective exposures.^[31]^ Based on the theoretical framework above and our analytical results, synergistic interactions were identified for the pairs of obesity and hypoalphalipoproteinemia, diabetes and hypertension, and diabetes and hypertriglyceridemia concerning their effect on reducing the clinical remission rate of IBD. This signifies that the simultaneous presence of these 2 components exerts a more detrimental effect on the remission rate than the sum of their individual effects (see Tables S15–S17, Supplemental Content 15–17, Supplemental Digital Content, https://links.lww.com/MD/R741, which illustrates the additive interaction between any pairwise combinations of metabolic syndrome components).

Binary logistic regression analysis revealed a significant inverse dose–response relationship between the number of metabolic syndrome components and the rate of clinical remission in IBD. In the unadjusted crude model, each additional metabolic syndrome component was associated with a 39.1% reduction in the odds of achieving clinical remission (OR = 0.609, 95% CI: 0.527–0.704, *P* = .001). After adjustment for age, smoking, alcohol consumption, baseline clinical activity of IBD, IBD duration, and IBD subtype, this inverse association remained significant and was slightly strengthened. Specifically, each additional metabolic syndrome component was associated with a 36.1% lower odds of clinical remission (adjusted OR = 0.639, 95% CI: 0.542–0.754, *P* < .001; see Table S22, Supplemental Content 22, Supplemental Digital Content, https://links.lww.com/MD/R741, which illustrates the binary logistic regression analysis of the association between the number of metabolic syndrome components and clinical remission in IBD).

Model fit statistics indicated that the adjusted model (model 2), which included the number of metabolic syndrome components and all covariates, was significantly superior to the crude model (model 1) containing only the number of metabolic syndrome components. The Nagelkerke *R*^2^ of the adjusted model was 0.180, indicating that it explained 18.0% of the variance in IBD clinical remission rates. This represents a 78% improvement in explanatory power compared to the crude model (*R*^2^ = 0.101). The likelihood ratio test was highly significant (*P* < .001) for both models, confirming their overall validity. Collectively, these results demonstrate a robust and independent inverse association between the number of metabolic syndrome components and the clinical remission rate in IBD (see Table S22, Supplemental Content 22, Supplemental Digital Content, https://links.lww.com/MD/R741, which illustrates the binary logistic regression analysis of the association between the number of metabolic syndrome components and clinical remission in IBD).

### 3.8. PSM analysis for confounding adjustment

**Figure 2.**
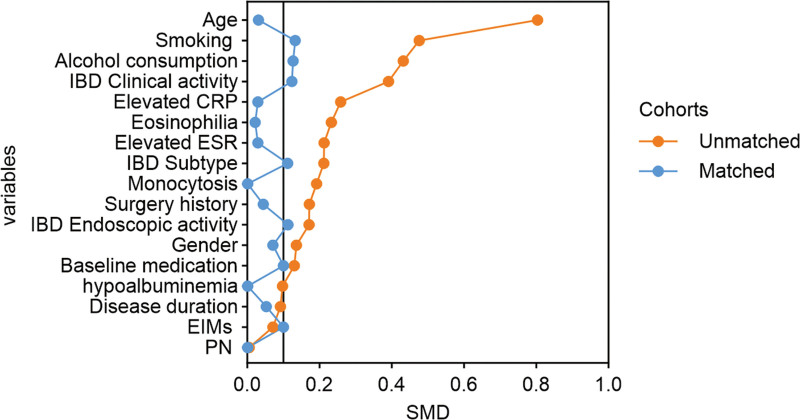
Dot plots showing SMD of baseline variables before and after PSM. IBD; CRP; ESR; EIMs; PN; the SMD values before (orange line) and after (blue line) propensity score matching are connected. Post-matching SMDs for all covariates were closely distributed on either side of the 0.1 threshold. CRP = C-reactive protein, EIM = extraintestinal manifestation, ESR = erythrocyte sedimentation rate, IBD = inflammatory bowel disease, PN = parenteral nutrition, PSM = propensity score matching, SMD = standardized mean difference.

**Figure 3.**
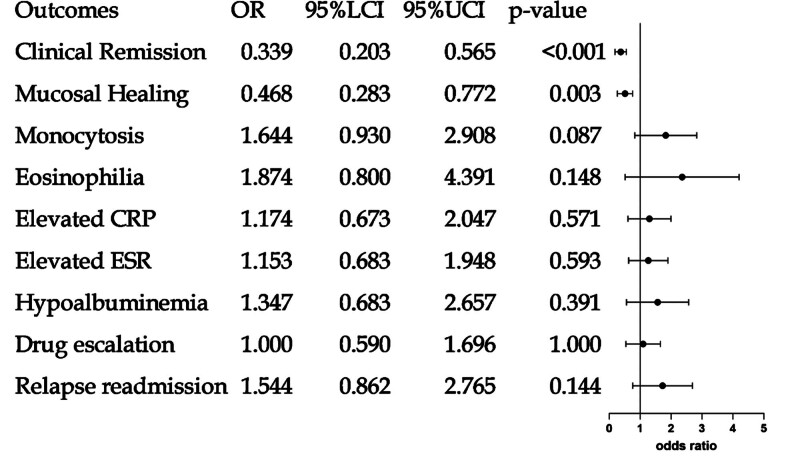
Plots of adjusted ORs of outcome of IBD (MetS vs non-MetS) after PSM. CRP, ESR; forest plot showing adjusted odds ratios (ORs) for outcomes in IBD patients with metabolic syndrome (MetS) versus those without (non-MetS), after propensity score matching. A significant protective association (OR < 1) was found specifically for clinical remission and mucosal healing. No significant associations were observed for the other presented outcomes. CRP = C-reactive protein, IBD = inflammatory bowel disease, MetS = metabolic syndrome, OR = odds ratio, PSM = propensity score matching.

**Figure 4.**
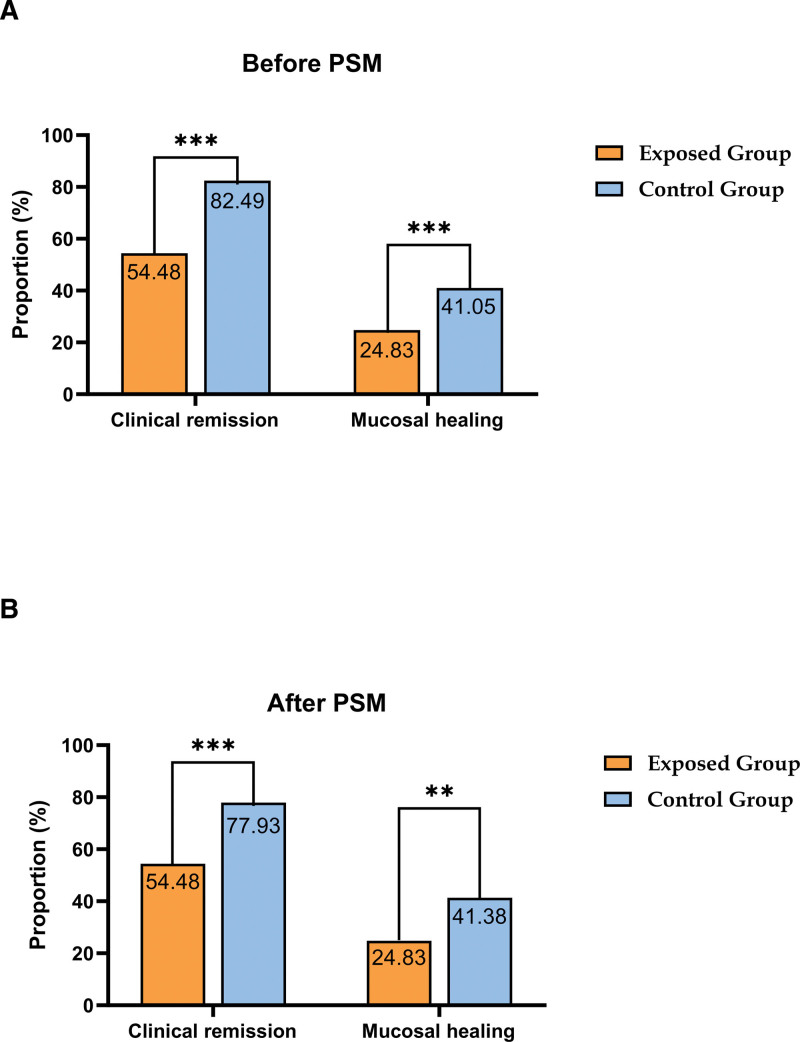
Rates of clinical remission and mucosal healing before and after propensity score matching. (A) Comparison before PSM of clinical remission and mucosal healing rates between the exposed group (IBD with MetS, orange) and control group (IBD without MetS, blue). (B) Comparison after PSM of the two outcomes in the matched cohorts. The statistical significance of the inter-group comparisons is marked: ***P < .001; **P < .01. IBD = inflammatory bowel disease, MetS = metabolic syndrome, PSM = propensity score matching.

We used PSM to adjust for baseline demographic and disease characteristics to generate matched cohorts of the IBD MetS and IBD groups (n = 290; see Table S23, Supplemental Content 23, Supplemental Digital Content, https://links.lww.com/MD/R741, which illustrates the baseline characteristics of IBD patients after PSM).. All variables except MetS were balanced to establish well-matched cohorts. The standardized mean difference plot was generated to evaluate covariate balance following PSM (Fig. 2). Relative to controls, IBD MetS patients had significantly reduced clinical remission rates (54.48% vs 77.93 %, OR: 0.339, 95% CI: 0.203–0.565; *P* < .001) and mucosal healing rates (24.83% vs 41.38%, OR: 0.468, 95% CI: 0.283–0.772; *P* = .003), increased frequencies of monocytosis (25.52% vs 17.24%, OR: 1.644, 95% CI: 0.930–2.908; *P* = .087), eosinophilia (11.03% vs 6.21%, OR: 1.874, 95% CI: 0.80–4.391; *P* = .148), elevated CRP (23.45% vs 20.69%, OR: 1.174, 95% CI: 0.673–2.047; *P* = .571), elevated ESR (27.59% vs 24.83%, OR: 1.153, 95% CI: 0.683–1.948; *P* = .593) and hypoalbuminemia (15.17% vs 11.72%, OR: 1.347, 95% CI: 0.683–2.657; *P* = .391), and higher IBD relapse-driven readmissions (23.45% vs 16.55%, OR: 1.544, 95% CI: 0.862–2.765; *P* = .144; Table 5, Fig. 3; see Table S24, Supplemental Content 24, Supplemental Digital Content, https://links.lww.com/MD/R741, which illustrates the odds ratios for disease activity and hospitalization outcomes of IBD patients at 12-months’ follow-up, after PSM).. Of Following matching, differences between the cohorts in factors such as monocytosis, eosinophilia, elevated CRP/ESR, hypoalbuminemia, medical expenses, relapse readmission, and surgical treatment for IBD were no longer statistically significant, in contrast to the pre-matching findings. Nevertheless, after the application of matching to control for potential confounders, the exposed group persistently exhibited significantly reduced rates of clinical remission and mucosal healing, compared to the control group (Fig. 4). These associations persisted with statistical significance, thereby confirming the pre-matching observations.

**Table 5:** Disease activity and hospitalization outcomes assessed at 12 months in patients with inflammatory bowel disease after propensity score matching.

Variables	Total (n = 290)	IBD MetS (n = 145)	IBD (n = 145)	*P*-value
Clinical remission, n (%)	192 (66.21)	79 (54.48)	113 (77.93)	<.001
Mucosal healing, n (%)	96 (33.10)	36 (24.83)	60 (41.38)	.003
Monocytosis, n (%)	62 (21.38)	37 (25.52)	25 (17.24)	.086
Eosinophilia, n (%)	25 (8.62)	16 (11.03)	9 (6.21)	.143
Elevated CRP, n (%)	64 (22.07)	34 (23.45)	30 (20.69)	.571
Elevated ESR, n (%)	76 (26.21)	40 (27.59)	36 (24.83)	.593
Hypoalbuminemia, n (%)	39 (13.45)	22 (15.17)	17 (11.72)	.389
Outpatient visit, M (*Q*_1_, *Q*_3_)	4.00 (2.0, 6.0)	4.00 (3.0, 6.0)	4.00 (2.0, 6.0)	.014
Medical expenses, M (*Q*_1_, *Q*_3_)	11,183.50 (5623.75, 22,208.75)	11,514.0 (6029.50, 22,345.0)	11,137.00 (5422.0, 21,444.0)	.857
Drug escalation, n (%)	74 (25.52)	37 (25.52)	37 (25.52)	1.000
Relapse readmission, n (%)	58 (20.00)	34 (23.45)	24 (16.55)	.142
Surgical treatment for IBD, n (%)	9 (3.10)	6 (4.14)	3 (2.07)	.498
Extraintestinal manifestations, n (%)	2 (0.69)	2 (1.38)	0 (0.0)	.478
Complication, n (%)	2 (0.69)	2 (1.38)	0 (0.0)	.478

Categorical variables are presented as n (%); continuous variables are presented as median (first quartile, third quartile).

CRP = C-reactive protein, ESR = erythrocyte sedimentation rate, IBD = inflammatory bowel disease, MetS = metabolic syndrome.

## 4. Discussion

The global incidence of both IBD and MetS has been rising at varying rates, paralleling the widespread adoption of high-fat diets and sedentary lifestyles. These 2 diseases share multiple common features as chronic, lifelong disorders. Growing evidence elucidates connections between them, including shared gene co-expression patterns^[12]^ and common pathogenic drivers such as immune dysregulation, chronic low-grade inflammation, insulin resistance, and gut microbiota alterations.^[11]^ This study investigated the impact of MetS on disease activity in IBD.

In our cohort of 1730 Chinese IBD patients, 156 (9%) were diagnosed with MetS. This prevalence is markedly lower than the reported global range of 10.6–32.7%.^[10]^ Several factors may account for this discrepancy. First, demographic and genetic differences likely play a role. Although our single-center study had a substantial sample size, its generalizability may be limited. Our cohort exclusively comprised Chinese individuals, who may have distinct genetic predispositions and lifestyle factors compared to the predominantly Western populations in studies establishing the global range. Second, methodological variations could be a contributing factor. Differences in the definitions of MetS, as well as the age and disease characteristics of the study populations, can significantly influence prevalence estimates. Further longitudinal and multi-center studies in our region are warranted to validate these observations and to better elucidate the interplay between IBD and MetS in this specific population.

In the baseline characteristics, IBD patients with MetS (IBD-MetS) were more likely to exhibit clinical activity than those with IBD alone and had higher levels of inflammatory biomarkers (monocytosis, eosinophilia, CRP, and ESR). After 12 months of follow-up, the IBD-MetS group had lower rates of clinical remission and mucosal healing, along with a higher likelihood of elevated inflammatory markers, compared to the IBD-only group. These findings persisted after adjustment for potential confounders, suggesting that MetS is associated with increased disease activity in IBD.

To further control for confounding, we used propensity score matching (PSM). After matching, IBD-MetS patients still had lower clinical remission and mucosal healing rates than those with IBD alone, indicating that MetS independently influences IBD disease activity. However, PSM nullified the significant differences in monocytosis, eosinophilia, elevated CRP/ESR, and hypoalbuminemia. While the reduced sample size after matching may have diminished statistical power, this result primarily indicates that the apparent association between MetS and these inflammatory indicators in the unmatched analysis was largely confounded by factors such as age, smoking, alcohol consumption, and clinical disease activity of IBD at baseline. Crucially, the significantly lower clinical remission rate in the MetS group persisted even after controlling for these variables. Consequently, the adverse impact of MetS on IBD outcomes is not mediated solely by systemic inflammation but likely involves other mechanisms, such as metabolic dysfunction, gut microbiota shifts, impaired tissue repair, or altered drug pharmacokinetics in obesity.

Our research points to an association between MetS and worse IBD activity. Interestingly, however, IBD-MetS patients did not receive escalated therapy with immunomodulators or biologics more frequently than the non-MetS group. This finding may reflect limitations inherent to retrospective cohort studies, such as potential selection bias. Previous studies suggest that obesity promote accelerated biologic clearance, resulting in poorer response to biologic therapy in IBD patients with obesity.^[32]^ After 12-months’ follow-up, the IBD-MetS group had significantly more annual outpatient visits, higher healthcare expenditures, elevated readmission rates, and greater surgical intervention frequency than the non-MetS group. However, after PSM, only the difference in outpatient visit frequency remained significant, while other healthcare utilization metrics and clinical outcomes showed no intergroup differences. Existing evidence suggests that obesity correlates with elevated readmission risk, prolonged hospitalization duration and greater inpatient expenditures among individuals with IBD.^[33]^ Patients with both diabetes and IBD have higher rates of adverse outcomes, complications, surgeries, and sepsis than those with IBD alone.^[34,35]^ The loss of significance for some metrics after PSM may be due to reduced statistical power following sample size reduction during matching.

The association between MetS and increased IBD activity likely stems from shared immunometabolic pathways. Genetic studies have identified shared molecular mechanisms.^[36]^ *HOXC4* modulates immune and metabolic processes through epigenetic (e.g., methylation) and transforming growth factor-β signaling pathways.^[36]^ Lipoprotein lipase (LPL) dysfunction, by promoting the accumulation of free fatty acids and oxidized low-density lipoprotein, activates TLR4/NF-κB and NLRP3 pathways to drive pro-inflammatory cytokine release, thereby positioning *LPL* as a critical link between lipid metabolism and chronic inflammation and a promising therapeutic target for the IBD-MetS comorbidity.^[36–38]^ Protein–Protein Interaction network analyses have identified several genes co-expressed in CD and MetS, including *EP300*, *RAC2*, *PIM2*, *PBX2*, and *PRKACA*, which are critically involved in metabolic regulation, immune response, and inflammation.^[12]^

Gut microbiota dysbiosis also plays central roles in IBD and MetS. Gut dysbiosis contributes to IBD pathogenesis through disease-specific pathways: in CD, it promotes macrophage-derived, tumor necrosis factor-α (TNF-α)-driven inflammation, whereas in UC, it elicits a dysregulated immune response featuring interleukin (IL)-13-mediated epithelial cytotoxicity and barrier disruption.^[3,4]^ Gut dysbiosis and intestinal barrier dysfunction are also recognized as key players in the initiation and advancement of metabolic syndrome.^[39]^

Adipose tissue itself is the pivotal inflammatory nexus shared by MetS and IBD. Central obesity, the hallmark initiator of MetS, drives insulin resistance and systemic inflammation via adipose-derived mediators.^[40]^ In obesity, immune cell infiltration into expanding adipose tissue promotes a chronic low-grade inflammatory state through mechanisms involving adipocyte stress, macrophage activation, and altered cytokine production. This creates a pathogenic cascade leading to glucose dysregulation and endothelial dysfunction.^[38]^ MetS is characterized by a pro-inflammatory adipokine profile (e.g., elevated leptin, resistin, TNF-α, IL-6, and decreased adiponectin) and a graded increase in systemic inflammation (e.g., CRP, TNF-α, IL-18) with accumulating metabolic components.^[40–50]^ Similarly, in IBD − particularly Crohn’s disease − hypertrophic mesenteric adipose tissue (“creeping fat”) actively secretes proinflammatory mediators, which can exacerbate intestinal inflammation and barrier damage.^[51–53]^ Thus, dysfunctional adipose tissue, via overlapping immunometabolic pathways, provides a direct mechanistic link through which MetS is likely associated with increased disease activity in IBD − a conclusion supported by our clinical observations.

Our preliminary findings suggest an association between MetS and increased disease activity in IBD, leading us to propose several management considerations for patients with this comorbidity. A Mediterranean diet rich in antioxidants and anti-inflammatory components, such as olive oil, fish, whole grains, vegetables and fruits, is recommended for IBD patients with MetS.^[54]^ Since both conditions are linked to gut microbiota alterations, a low-fat, high-fiber diet may help support microbial balance.^[55–57]^ In the context of modern environmental influences, such as poor dietary habits and sedentary lifestyles, the holistic impact on the body has garnered increasing attention. Thus, beyond addressing individual components of MetS in relation to IBD, we must prioritize understanding the systemic effects of MetS as an integrated entity. This includes comprehensive monitoring of weight, blood pressure, blood glucose, and lipid levels, coupled with targeted interventions to control hypertension, hyperglycemia, dyslipidemia, and body weight.^[40]^

Notwithstanding adjustments for confounders via multivariate analysis and PSM, this study has several limitations inherent to its retrospective design. Dependence on medical records and patient recall may introduce information gaps, recording biases, and outcome misclassification. Specifically, waist circumference data were unavailable, leading to the use of BMI as a surrogate for central obesity, which might have affected the accuracy of MetS identification. Furthermore, although IBD duration was documented, the onset of MetS could not be precisely determined due to low patient awareness of its components and incomplete electronic health records prior to 2013. Consequently, the temporal relationship between IBD and MetS remains unclear. While our results suggest an association between MetS and increased IBD disease activity, causality cannot be inferred. Additionally, although medication usage was recorded at baseline and at 12 months, the potential impact of adherence on outcomes cannot be fully excluded. Thus, further prospective studies are warranted to validate these findings.

## 5. Conclusions

In recent years, the incidence of IBD complicated with metabolic syndrome (MetS) has been increasing annually. We conducted a retrospective cohort study to explore the impact of metabolic syndrome on disease activity in IBD. Our results showed that compared to IBD alone, patients with IBD complicated by metabolic syndrome had lower rates of clinical remission and mucosal healing. After adjusting for confounding factors, the same results were observed. Therefore, we hypothesize that metabolic syndrome is associated with increased disease activity in IBD. This conclusion highlights the importance of paying attention to the metabolic status of IBD patients. Greater emphasis should be placed on the disease activity of patients with IBD complicated by metabolic syndrome, and careful diagnostic and therapeutic decisions should be made accordingly.

## Acknowledgments

The authors have no acknowledgments to declare.

## Author contributions

**Conceptualization:** Pingping Liu, Xue Jing, Xueli Ding, Beibei Ma.

**Data curation:** Pingping Liu, Xue Jing, Lingling Sun, Shijin Wang, Jingli Zhang, Lijuan Sun, Beibei Ma.

**Formal analysis:** Pingping Liu, Xue Jing, Lingling Sun, Shijin Wang, Jingli Zhang, Xueli Ding, Lijuan Sun, Beibei Ma.

**Funding acquisition:** Shijin Wang.

**Investigation:** Pingping Liu, Lingling Sun, Shijin Wang, Jingli Zhang, Xueli Ding, Lijuan Sun, Beibei Ma.

**Methodology:** Pingping Liu, Lingling Sun, Jingli Zhang, Xueli Ding, Beibei Ma.

**Supervision:** Xue Jing, Lingling Sun, Shijin Wang, Jingli Zhang, Xueli Ding, Lijuan Sun, Beibei Ma.

**Validation:** Jingli Zhang.

**Visualization:** Lingling Sun, Shijin Wang.

**Writing – original draft:** Pingping Liu.

**Writing – review & editing:** Xue Jing, Lingling Sun, Beibei Ma.

## Supplementary Material


