# Supplementary material for: Influence of metabolic syndrome on disease characteristics and activity in inflammatory bowel disease: A retrospective cohort study
Source: Medicine (Baltimore). 2026 Apr 24;105(17):e48426. doi: 10.1097/MD.0000000000048426 (PMC13124333; doi:10.1097/MD.0000000000048426)
Supplement: Supplementary file 1 [file medi-105-e48426-s001.pdf]

Supplementary Table 1. Healthcare resource utilization and clinical event outcomes at 12-months' follow-up.

Variables	Total (n = 676)	IBD MetS (n = 145)	IBD (n = 531)	P-value
Yearly outpatient visits, M (Q_1, Q_3)	4.0 (3.0, 6.0)	4. (3.00, 6.0)	4.0 (3.0, 6.0)	.035
Medical expenses (CNY), M (Q_1, Q_3)	9857.50 (4613.75,19 832.50)	11514.0 (6029.50,22 345.0)	9176.0 (4201.0,18745.50)	.029
Drug escalation, n (%)	154 (22.78)	37 (25.52)	117 (22.03)	.375
Relapse readmission, n (%)	88 (13.02)	34 (23.45)	54 (10.17)	<.001
Surgical treatment for IBD, n (%)	12 (1.78)	6 (4.14)	6 (1.13)	.038
Extraintestinal manifestations A, n (%)	3 (0.44)	2 (1.38)	1 (0.19)	.118
Complication, n (%)	5 (0.74)	2 (1.38)	3 (0.56)	.640

CNY = Chinese Yuan. Data are presented as Median (First Quartile, Third Quartile).

Supplementary Table 2.Association of Obesity and Diabetes with Clinical remission rate, and Their Multiplicative Interaction

Variable	B	Std. Error	P	*OR (95%CI)*
Obesity	-0.418	0.254	.099	0.658(0.400,1.082)
Diabetes	-1.038	0.253	.001	0.354(0.216,0.581)
Obesity*Diabetes	0.154	0.399	.699	1.167(0.533,2.552)

B = unstandardized regression coefficient, Std. Error = standard error, OR = odds ratio, CI = confidence interval.

Supplementary Table 3.Association of Obesity and Hypertension with Clinical remission rate, and Their Multiplicative Interaction

Variable	B	Std. Error	P	OR（95%CI）
Obesity	-0.404	0.240	.093	0.668 (0.417, 1.069)
Hypertension	-0.970	0.324	.003	0.379(0.201, 0.714)
Obesity*Hypertension	0.371	0.446	.406	1.449(0.605, 3.471)

B = unstandardized regression coefficient, Std. Error = standard error, OR = odds ratio, CI = confidence interval.

Supplementary Table 4.Association of Obesity and Hypertriglyceridemia with Clinical remission rate, and Their Multiplicative Interaction

Variable	B	Std. Error	P	*OR （95%CI）*
Obesity	-0.431	0.240	.073	0.650(0.406, 1.041)
Hypertriglyceridemia	-1.149	0.273	.001	0.317(0.186, 0.542)
Obesity*Hypertriglyceridemia	0.2539	0.417	.542	1.289(0.570, 2.916)

B = unstandardized regression coefficient, Std. Error = standard error, OR = odds ratio, CI = confidence interval.

Supplementary Table 5.Association of Obesity and Hypoalphalipoproteinemia with Clinical remission rate, and Their Multiplicative Interaction

Variable	B	Std. Error	P	*OR (95%CI)*
Obesity	-0.700	0.227	.002	0.497(0.319,0.774)
Hypoalphalipoproteinemia	-0.606	0.243	.013	0.546(0.339, 0.878)
Obesity*Hypoalphalipoproteinemia	0.539	0.419	.198	1.715(0.754,3.900)

B = unstandardized regression coefficient, Std. Error = standard error, OR = odds ratio, CI = confidence interval.

Supplementary Table 6.Association of Diabetes and Hypertension with Clinical remission rate, and Their Multiplicative Interaction

Variable	B	Std. Error	P	*OR (95%CI)*
Diabetes	-1.065	0.243	.001	0.345(0.214, 0.554)
Hypertension	-0.906	0.322	.005	0.404(0.215, 0.760)
Diabetes*Hypertension	0.684	0.447	.126	1.981(0.825, 4.757)

B = unstandardized regression coefficient, Std. Error = standard error, OR = odds ratio, CI = confidence interval.

Supplementary Table 7.Association of Diabetes and Hypertriglyceridemia with Clinical remission rate, and Their Multiplicative Interaction

Variable	B	Std. Error	P	*OR（95%CI）*
Diabetes	-1.020	0.245	.001	0.360(0.223,0.582)
Hypertriglyceridemia	-1.135	0.280	.001	0.321(0.186,0.556)
Diabetes*Hypertriglyceridemia	0.543	0.421	.197	1.721(0.754,3.924)

B = unstandardized regression coefficient, Std. Error = standard error, OR = odds ratio, CI = confidence interval.

Supplementary Table 8.Association of Diabetes and Hypoalphalipoproteinemia with Clinical remission rate, and Their Multiplicative Interaction

Variable	B	Std. Error	P	*OR（95%CI）*
Diabetes	-1.044	0.231	.001	0.352(0.224, 0.554)
Hypoalphalipoproteinemia	-0.426	0.249	.088	0.653(0.401,1.065)
Diabetes*Hypoalphalipoproteinemia	-0.039	0.425	.927	0.962(0.418,2.212)

B = unstandardized regression coefficient, Std. Error = standard error, OR = odds ratio, CI = confidence interval.

Supplementary Table 9.Association of Hypertension and Hypertriglyceridemia with Clinical remission rate, and Their Multiplicative Interaction

Variable	B	Std. Error	P	*OR (95%CI)*
Hypertension	-0.424	0.313	0.175	0.655(0.355, 1.208)
Hypertriglyceridemia	-0.829	0.269	0.002	0.436(0.258,0.739)
Hypertension*Hypertriglyceridemia	-0.300	0.466	0.520	0.741(0.297, 1.846)

Abbreviations: B, unstandardized regression coefficient; Std. Error, standard error; OR, odds ratio; CI, confidence interval.

Supplementary Table 10.Association of Hypertension and Hypoalphalipoproteinemia with Clinical remission rate, and Their Multiplicative Interaction

Variable	B	Std. Error	P	*OR (95%CI)*
Hypertension	-0.828	0.250	0.001	0.437(0.267, 0.714)
Hypoalphalipoproteinemia	-0.374	0.230	0.103	0.688(0.439, 1.079)
Hypertension*Hypoalphalipoproteinemia	-0.289	0.473	0.542	0.749(0.297, 1.893)

Abbreviations: B, unstandardized regression coefficient; Std. Error, standard error; OR, odds ratio; CI, confidence interval.

Supplementary Table 11.Association of Hypertriglyceridemia and Hypoalphalipoproteinemia with Clinical remission rate, and Their Multiplicative Interaction

Variable	B	Std. Error	P	*OR (95%CI)*
Hypertriglyceridemia	-1.115	0.249	0.001	0.328(0.201,0.534)
Hypoalphalipoproteinemia	-0.364	0.244	0.135	0.695(0.431, 1.1205)
Hypertriglyceridemia* Hypoalphalipoproteinemia	0.082	0.433	0.850	1.085(0.464,2.537)

Abbreviations: B, unstandardized regression coefficient; Std. Error, standard error; OR, odds ratio; CI, confidence interval.

Supplementary Table 12.Additive Interaction between Obesity and Diabetes on Clinical Remission Rate

Obesity	Diabetes	*P*	*OR* (95%CI)	*RERI* (95% CI)	*AP* (95% CI)	*S* (95% CI)
NO	NO	-	1.00(Reference)	-	-	-
YES	NO	.099	0.658(0.400,1.082)	-	-	-
NO	YES	<.001	0.354(0.216,0.581)	-	-	-
YES	YES	<.001	0.272(0.162,0.457)	0.260 (-0.130, 0.649)	0.955 (-0.567, 2.476)	0.737 (0.496 ,1.096)

RERI = relative excess risk due to interaction, AP = attributable proportion due to interaction, S = synergy index. All measures assess additive interaction.

Supplementary Table 13.Additive Interaction between Obesity and Hypertension on Clinical Remission Rate

Obesity	Hypertension	*P*	*OR* (95%CI)	*RERI* (95% CI)	*AP* (95% CI)	*S* (95% CI)
NO	NO	-	1.00(Reference)	-	-	-
YES	NO	.093	0.668(0.417, 1.069)	-	-	-
NO	YES	.003	0.379(0.201, 0.714)	-	-	-
YES	YES	<.001	0.367(0.220,0.611)	0.320 (-0.100, 0.739)	0.872 (-0.290, 2.034)	0.664 (0.417, 1.058)

RERI = relative excess risk due to interaction, AP = attributable proportion due to interaction, S = synergy index. All measures assess additive interaction.

Supplementary Table 14.Additive Interaction between Obesity and Hypertriglyceridemia on Clinical Remission Rate

Obesity	Hypertriglyceridemia	P	OR (95%CI)	RERI (95% CI)	AP (95% CI)	S (95% CI)
NO	NO	-	1.00 (Reference)	-	-	-
YES	NO	.073	0.650 (0.406, 1.041)	-	-	-
NO	YES	<.001	0.317 (0.186, 0.542)	-	-	-
YES	YES	<.001	0.266 (0.154,0.457)	0.299 (-0.071, 0.668)	1.125 (-0.362, 2.611)	S=0.711 (0.493, 1.024)

RERI = relative excess risk due to interaction, AP = attributable proportion due to interaction, S = synergy index. All measures assess additive interaction.

Supplementary Table 15.Additive Interaction between Obesity and Hypoalphalipoproteinemia on Clinical Remission Rate

Obesity	Hypoalphalipoproteinemia	*P*	*OR* (95%CI)	*RERI* (95% CI)	*AP* (95% CI)	*S* (95% CI)
NO	NO	-	1.00 (Reference)	-	-	-
YES	NO	.002	0.497 (0.319,0.774)	-	-	-
NO	YES	.013	0.546 (0.339,0.878)	-	-	-
YES	YES	.019	0.465 (0.245,0.880)	0.422 (0.009,0.836)	0.909 (0.070,1.748)	0.559 (0.314,0.995)

RERI = relative excess risk due to interaction, AP = attributable proportion due to interaction, S = synergy index. All measures assess additive interaction.

Supplementary Table 16.Additive Interaction between Diabetes and Hypertension on Clinical Remission Rate

Diabetes	Hypertension	*P*	*OR* (95%CI)	*RERI* (95% CI)	*AP* (95% CI)	*S* (95% CI)
NO	NO	-	1.00 (Reference)	-	-	-
YES	NO	<.001	0.345 (0.214,0.554)	-	-	-
NO	YES	.005	0.404 (0.215, 0.760)	-	-	-
YES	YES	<.001	0.276 (0.164,0.463)	0.527 (0.205,0.850)	1.910 (0.507, 3.314)	0.579 (0.436,0.768)

RERI = relative excess risk due to interaction, AP = attributable proportion due to interaction, S = synergy index. All measures assess additive interaction.

Supplementary Table 17.Additive Interaction between Diabetes and Hypertriglyceridemia on Clinical Remission Rate

Diabetes	Hypertriglyceridemia	*P*	*OR* (95%CI)	*RERI* (95% CI)	*AP* (95% CI)	*S* (95% CI)
NO	NO	-	1.00 (Reference)	-	-	-
YES	NO	<.001	0.360 (0.223,0.582)	-	-	-
NO	YES	<.001	0.321 (0.186, 0.556)	-	-	-
YES	YES	<.001	0.199 (0.116,0.342)	0.517 (0.254,0.781)	2.596 (0.737, 4.456)	0.607 (0.492,0.750)

RERI = relative excess risk due to interaction, AP = attributable proportion due to interaction, S = synergy index. All measures assess additive interaction.

Supplementary Table 18.Additive Interaction between Diabetes and Hypoalphalipoproteinemia on Clinical Remission Rate

Diabetes	Hypoalphalipoproteinemia	*P*	*OR* (95%CI)	*RERI* (95% CI)	*AP* (95% CI)	*S* (95% CI)
NO	NO	-	1.00 (Reference)	-	-	-
YES	NO	<.001	0.352 (0.224,0.554)	-	-	-
NO	YES	.088	0.653 (0.401, 1.065)	-	-	-
YES	YES	<.001	0.221 (0.117,0.418)	0.216 (0.169,0.601)	0.976 (-0.844, 2.796)	0.783 (0.530,1.158)

RERI = relative excess risk due to interaction, AP = attributable proportion due to interaction, S = synergy index. All measures assess additive interaction.

Supplementary Table 19.Additive Interaction between Hypertension and Hypertriglyceridemia on Clinical Remission Rate

Hypertension	Hypertriglyceridemia	*P*	*OR* (95%CI)	*RERI* (95% CI)	*AP* (95% CI)	*S* (95% CI)
NO	NO	-	1.00 (Reference)	-	-	-
YES	NO	.175	0.655 (0.355,1.208)	-	-	-
NO	YES	.002	0.436 (0.258,0.739)	-	-	-
YES	YES	<.001	0.212 (0.123,0.363)	0.121 (-0.36,0.603)	0.570 (-1.731, 2.871)	0.867 (0.510, 1.476)

RERI = relative excess risk due to interaction, AP = attributable proportion due to interaction, S = synergy index. All measures assess additive interaction.

Supplementary Table 20.Additive Interaction between Hypertension and Hypoalphalipoproteinemia on Clinical Remission Rate

Hypertension	Hypoalphalipoproteinemia	*P*	*OR* *(95%CI)*	*RERI* *(95% CI)*	*AP* *(95% CI)*	*S* *(95% CI)*
NO	NO	-	1.00 (Reference)	-	-	-
YES	NO	<.001	0.437 (0.267,0.714)	-	-	-
NO	YES	.103	0.688 (0.439,1.079)	-	-	-
YES	YES	<.001	0.225 (0.108,0.471)	0.100 (-0.319, 0.519)	0.445 (-1.390, 2.280)	0.885 (0.546,1.436)

RERI = relative excess risk due to interaction, AP = attributable proportion due to interaction, S = synergy index. All measures assess additive interaction.

Supplementary Table 21.Additive Interaction between Hypertriglyceridemia and Hypoalphalipoproteinemia on Clinical Remission Rate

Hypertriglyceridemia	Hypoalphalipoproteinemia	*P*	*OR* (95%CI)	*RERI* (95% CI)	*AP* (95% CI)	*S* (95% CI)
NO	NO	-	1.00 (Reference)	-	-	-
YES	NO	<.001	0.328 (0.201,0.534)	-	-	-
NO	YES	.135	0.695 (0.431,1.1205)	-	-	-
YES	YES	<.001	0.247 (0.132,0.461)	0.225 (-0.171, 0.620)	0.910 (-0.728, 2.546)	0.770 (0.510, 1.163)

RERI = relative excess risk due to interaction, AP = attributable proportion due to interaction, S = synergy index. All measures assess additive interaction.

Supplementary Table 22.Binary Logistic Regression Analysis of the Association between the Number of Metabolic Syndrome Components and Clinical Remission in IBD

Variable	Model 1: Crude Model		Model 2: Adjusted Model	
	OR (95% CI)	P-value	aOR (95% CI)	P-value
Number of MetS Components (per 1-component increase)	0.609(0.527,0.704)	.001	0.639(0.542,0.754)	<.001
Adjusted Covariates				
Age (per 1-year increase)	-	-	1.001(0.987,1.015)	.933
Smoking	-	-	0.681(0.331,1.402)	.297
Alcohol consumption	-	-	2.162(1.024,4.566)	.043
Baseline clinical activity of IBD	-	-	0.570(0.462,0.705)	<.001
IBD duration (per 1-year increase)	-	-	0.975(0.940,1.010)	.163
IBD subtype	-	-	0.940(0.768,1.149)	.545
Model Fit Statistics				
Sample Size, n	676		676	
-2 Log Likelihood	690.439		651.325	
Likelihood Ratio Test P-value	P<0.001		P<0.001	
Nagelkerke R^2	0.101		0.180	

CI = confidence interval, OR = odds ratio, aOR = adjusted odds ratio, MetS = metabolic syndrome, IBD = inflammatory bowel disease. Model 1 is unadjusted. Model 2 is adjusted for all covariates listed in the table. Data for continuous variables are expressed.

Supplementary Table 23.Baseline characteristics of IBD patients after PSM

Variable	IBD MetS (n = 145)	IBD (n = 145)	P	SMD
Age, mean ± SD	58.14 ± 12.20	57.76 ± 12.68	.792	0.031
Gender, n (%)			.631	0.071
Female	55 (37.9)	60 (41.4)		
Male	90 (62.1)	85 (58.6)		
Smoking, n (%)	36 (24.8)	28 (19.3)	.322	0.133
Alcohol consumption, n (%)	29 (20.0)	22 (15.2)	.355	0.127
Disease characteristics				
Disease duration(y), mean ± SD	4.12 ± 5.27	4.42 ± 6.13	.651	0.053
IBD subtype, n (%)			.825	0.112
E1	51 (35.2)	45 (31.0)		
E2	22 (15.2)	20 (13.8)		
E3	58 (40.0)	65 (44.8)		
CD	14 (9.7)	15 (10.3)		
Parenteral nutrition, n (%)	2 (1.4)	2 (1.4)	1.000	<0.001
Extraintestinal manifestations, n (%)	5 (3.4)	8 (5.5)	.570	0.100
Baseline medications, n (%)			.868	0.100
No medication history	46 (31.7)	42 (29.0)		
5-aminosalicylic acids	78 (53.8)	85 (58.6)		

Variable	IBD MetS (n = 145)	IBD (n = 145)	P	SMD
Hormonal agents	9 (6.2)	8 (5.5)		
Immunomodulators or biologics	12 (8.3)	10 (6.9)		
Surgery, n (%)	4 (2.8)	3 (2.1)	1.000	0.045
IBD clinical activity, n (%)			.776	0.124
Normal or remission	26 (17.9)	31 (21.4)		
Mild	50 (34.5)	51 (35.2)		
Moderate	42 (29.0)	35 (24.1)		
Severe	27 (18.6)	28 (19.3)		
IBD endoscopic activity, n (%)			.820	0.113
Normal or remission	9 (6.2)	12 (8.3)		
Mild	28 (19.3)	25 (17.2)		
Moderate	72 (49.7)	68 (46.9)		
Severe	36 (24.8)	40 (27.6)		
Biomarkers of activity				
Hypoalbuminemia, n (%)	31 (21.4)	31 (21.4)	1.000	<0.001
Monocytosis, n (%)	33 (22.8)	33 (22.8)	1.000	<0.001
Eosinophilia, n (%)	17 (11.7)	16 (11.0)	1.000	0.022
Elevated CRP, n (%)	42 (29.0)	44 (30.3)	.898	0.030
Elevated ESR, n (%)	44 (30.3)	42 (29.0)	.898	0.030

IBD = inflammatory bowel disease, PSM = propensity score matching, MetS = metabolic syndrome, SMD = standardized mean difference, SD = standard deviation, UC = ulcerative colitis, CD = Crohn's

disease, CRP = C-reactive protein, ESR = erythrocyte sedimentation, *Montreal Classification for UC: E1 = proctitis, E2 = left-sided colitis, E3 = extensive colitis,*Continuous variables are presented as Mean ± SD, categorical variables are presented as n (%).

Supplementary Table 24.ORs for disease activity and hospitalization outcomes of IBD patients at 12-months' follow-up, after PSM

Outcomes	IBD MetS	IBD	OR (95% CI)	P
Clinical remission, n (%)	54.48	77.93	0.339 (0.203, 0.565)	<.001
Mucosal healing, n (%)	24.83	41.38	0.468 (0.283, 0.772)	.003
Monocytosis, n (%)	25.52	17.24	1.644 (0.930, 2.908)	.087
Eosinophilia, n (%)	11.03	6.21	1.874 (0.80, 4.391)	.148
Elevated CRP, n (%)	23.45	20.69	1.174 (0.673, 2.047)	.571
Elevated ESR, n (%)	27.59	24.83	1.153 (0.683, 1.948)	.593
Hypoalbuminemia, n (%)	15.17	11.72	1.347 (0.683, 2.657)	.391
Drug escalation, n (%)	25.52	25.52	1.0 (0.590, 1.696)	1.000
Relapse readmission, n (%)	23.45	16.55	1.544 (0.862, 2.765)	.144

IBD = inflammatory bowel disease, MetS = metabolic syndrome, OR = odds ratio, CI = confidence interval, CRP = C-reactive protein, ESR = erythrocyte sedimentation rate.
